# Head-to-Head Comparison of Different Blood Collecting Tubes for Quantification of Alzheimer’s Disease Biomarkers in Plasma

**DOI:** 10.3390/biom12091194

**Published:** 2022-08-28

**Authors:** Lijun Jiang, Xulong Ding, Wenxiao Wang, Xiaobin Yang, Tao Li, Peng Lei

**Affiliations:** 1 Mental Health Center and Department of Neurology, State Key Laboratory of Biotherapy, West China Hospital, Sichuan University, Chengdu 610041, China; 2 Dushu Lake Hospital Affiliated to Soochow University, Medical Center of Soochow University, Suzhou Dushu Lake Hospital, Suzhou 215125, China; 3 Deyang Mental Health Center, Deyang 618099, China; 4 Department of Neurobiology, Affiliated Mental Health Center & Hangzhou Seventh People’s Hospital, Zhejiang University School of Medicine, Hangzhou 310063, China; 5 NHC and CAMS Key Laboratory of Medical Neurobiology, MOE Frontier Science Center for Brain Science and Brain-machine Integration, School of Brain Science and Brain Medicine, Zhejiang University, Hangzhou 310012, China

**Keywords:** blood collection tubes, tau, Alzheimer’s disease, Simoa, EDTA, heparin

## Abstract

To examine whether the type of blood collection tubes affects the quantification of plasma biomarkers for Alzheimer’s disease analyzed with a single-molecule array (Simoa), we recruited a healthy cohort (n = 34, 11 males, mean age = 28.7 ± 7.55) and collected plasma in the following tubes: dipotassium ethylenediaminetetraacetic acid (K2-EDTA), heparin lithium (Li-Hep), and heparin sodium (Na-Hep). Plasma tau, phosphorylated tau 181 (p-tau181), amyloid β (1–40) (Aβ40), and amyloid β (1–42) (Aβ42) were quantified using Simoa. We compared the value of plasma analytes, as well as the effects of sex on the measurements. We found that plasma collected in Li-Hep and Na-Hep tubes yielded significantly higher tau and p-tau181 levels compared to plasma collected in K2-EDTA tubes from the same person, but there was no difference in the measured values of the Aβ40, Aβ42, and Aβ42/40 ratio. Therefore, the type of blood collecting tubes should be considered when planning studies that measure plasma tau.

## 1. Introduction

Blood-based biomarkers for neurodegenerative diseases are set to revolutionize geriatric medicine by significantly improving diagnostic accuracy in clinical settings [1]. Amyloid β (Aβ), total tau (tau), and tau phosphorylated at threonine 181 (p-tau181) have been widely studied. For example, plasma Aβ42/Aβ40 ratio may reflect Aβ pathology in the brain and demonstrate prediction of abnormal Aβ-PET outcomes with moderate accuracy [2]. Plasma tau and p-tau181 were found to be increased in Alzheimer’s disease (AD) specifically but not in any other neurodegenerative diseases [3,4]. In particular, plasma p-tau181 accurately differentiated individuals with AD neuropathology from those without, including those with non-AD tau pathology in a cohort of cognitive decline [5,6,7]. Therefore, when a critical amount of data has been collected, it is possible to establish worldwide reference levels for these parameters to diagnose AD [8]. However, such practice will require the standardization of the blood collection process between laboratories or hospitals worldwide, which is not sufficiently studied.

Blood collecting tubes (including dipotassium ethylenediaminetetraacetic acid [K2-EDTA], lithium/heparin [Li-Hep], sodium/heparin [Na-Hep], etc.) can affect the results of a variety of laboratory tests. For example, the types of blood tubes affect routine blood biochemistry results such as albumin [9], acetylcholinesterase [10], myoglobin [11], and hormone and tumor marker levels [12]. EDTA can competitively bind to calcium ions within the blood that is required for coagulation, therefore cannot be used in studies on metal ion biology [13]. Heparin, on the other hand, binds to antithrombin III and potentiates the inactivation of serine proteases of antithrombin III [14], and the heparin-coated tube is suitable for erythrocyte fragility tests, blood gas analysis, hematocrit [15], but unsuitable for polymerase chain reaction (PCR) reactions as heparin inhibits the Taq polymerase [16].

For studies on AD plasma biomarkers, EDTA tubes and heparin tubes are most commonly used [17,18,19,20,21]. As those tubes prevent blood coagulation via independent mechanisms, it is necessary to understand if they interact with targeted biological molecules such as Aβ and tau and therefore yield different results. Here, we have examined the influence of the type of blood collecting tubes on AD plasma markers in a cohort of young and healthy adults. We collected plasma from the same person using three different tubes (namely, K2-EDTA, Li-Hep, and Na-Hep), and measured total tau, p-tau181, Aβ 40, and Aβ 42 levels under the same condition using an ultrasensitive single molecule array (Simoa) [22]. We then compared the calculated values, the ratio changes, the data distribution, and their associations with gender.

## 2. Materials and Methods

### 2.1. Study Design and Participants

Thirty-four Chinese/Han participants were enrolled in 2 time periods, with the first population collection in November 2020, and the second in August 2021, with the following inclusion criteria: (1) age ≥ 20 years and ≤ 60 years; (2) education ≥ 16 years; (3) no difficulty in Chinese communication. Exclusion criteria were histories of neurological or psychiatric diseases and/or cognitive decline. The ethics committee of the Mental Health Center of Deyang City, Sichuan, China, approved all study protocols (identifier: 2018-116). Participants have provided written informed consent.

### 2.2. Blood Collection

Non-fasting venous blood was collected during the hours of 8 am–6 pm. Blood samples were collected by venipuncture and collected in K2-EDTA, Li-Hep, and Na-Hep tubes according to standard procedures. Blood samples were stood and centrifuged (2000 r/min, 10 min, room temperature), and the collected pure plasma was aliquoted and immediately stored at −80 °C.

### 2.3. Quantification of AD Biomarkers

Plasma tau, p-tau181, Aβ40, and Aβ42 were measured by immunoassay according to the protocols provided by the manufacturer, using a Simoa SR-X analyzer (Quanterix; Lexington, MA, USA). Specifically, in preparation for Alzheimer’s disease biomarkers quantification, samples were thawed and centrifuged at 2000× *g* for 5 min at 4 °C. Then, all samples were transferred to 96-well plates and diluted 4-fold in sample diluent, following a 2-step digital immunoassay, and 7 or 8 calibrators samples and 2 quality control samples were run on each plate for each analyte. The Simoa Neuro 3-Plex A kit (Cat #: 101995) was used to measure the levels of tau, Aβ40, and Aβ42, and the Simoa p-tau181 Advantage V2 Kit (Cat #: 103714) was used to measure the levels of p-tau181. The limit of detection for tau, p-tau181, Aβ40, and Aβ42 were 0.0165 pg/mL, 0.041 pg/mL, 0.243 pg/mL, 0.147 pg/mL, respectively; and the analytical ranges for tau, p-tau181, Aβ40 and Aβ42 were between 0 and 400 pg/mL, between 0 and 424 pg/mL, between 0 and 600 pg/mL, between 0 and 200 pg/mL, respectively.

### 2.4. Statistical Analyses

Data analyses were performed using R, version 3.3.1 (R Foundation) and GraphPad Prism 6 (GraphPad Software 6.0, San Diego, CA, USA). All hypothesis testing was 2-tailed, and paired independent t-tests or two-way ANOVA assessed the differences between groups for continuous variables, and the Kolmogorov–Smirnov test was used to test the equality between two densities. The data are expressed as the mean ± standard deviation (SD) for numerical variables or the count (%) for categorical variables. For all statistical tests, a *p*-value below 0.05 was considered statistically significant.

## 3. Results

### 3.1. Demographic and Clinical Characteristics

A total of 34 participants with signed consent were enrolled (Table 1), with no screening failures. The mean (SD) age of the participants was 28.71 (7.56) years old, 11 (32.4%) of whom were males. Meanwhile, the years of education of participants were greater than 16 years, with a mean (SD) of 18.38 (1.72).

### 3.2. Blood Collection Methods Affect the Results of Plasma Tau

**Figure 1:**
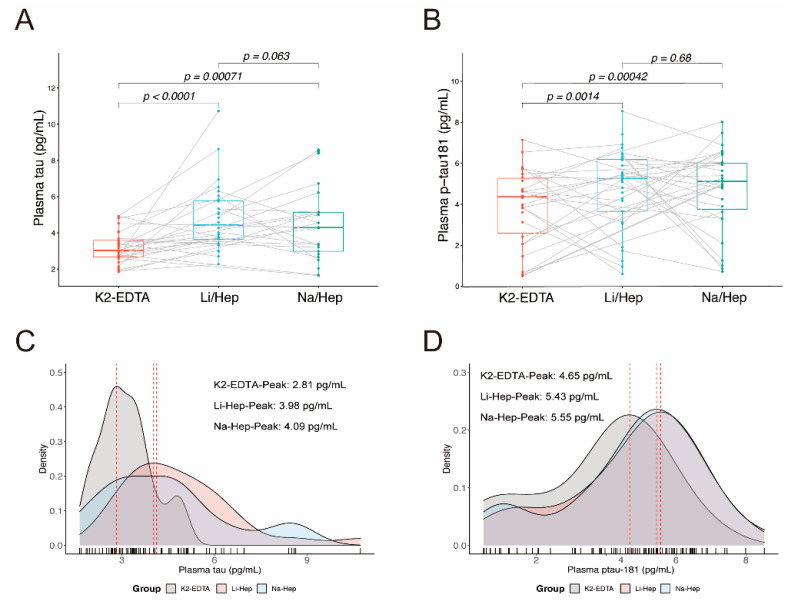
Blood collection methods affect the detection of tau and p-tau181 levels. (**A**,**B**) AD biomarkers comparison among K2-EDTA, Li-Hep, and Na-Hep tubes, including tau (**A**) and p-tau181 (**B**), Data were means ± SD; (**C**,**D**) Density plot of tau (**C**) and p-tau181 (**D**) among K2-EDTA, Li-Hep, and Na-Hep tubes. (**A**,**B**): The differences between groups were assessed by paired t-tests; (**C**,**D**): The peak levels were calculated and statistically compared using the Kolmogorov–Smirnov test. n was indicated in the methods and Table 1.

Plasma tau and p-tau181 collected in the K2-EDTA tube, Li-Hep tube, and Na-Hep tube were measured independently using pre-prepared Simoa kits from the same manufactory lot (Figure 1 and Table 1). The mean plasma tau and p-tau181 levels were significantly higher in the Li-Hep tube compared with the K2-EDTA tube (tau: K2-EDTA vs Li-Hep: 3.15 ± 0.86 vs 4.87 ± 1.85, *p* < 0.0001; p-tau181: K2-EDTA vs Li-Hep: 3.95 ± 1.86 vs 4.84 ± 1.95, *p* = 0.0014. Figure 1A,B). Similar results were observed when comparing K2-EDTA with Na-Hep, including significantly higher plasma tau and p-tau181 levels in the Na-Hep tube (tau: K2-EDTA vs Na-Hep: 3.15 ± 0.86 vs 4.41 ± 2.02, *p* = 0.00071; p-tau181: K2-EDTA vs Na-Hep: 3.95 ± 1.86 vs 4.78 ± 1.97, *p* = 0.0004. Figure 1A,B). The plasma values of tau and p-tau181 were not significantly different between Li-Hep and Na-Hep groups (tau: *p* = 0.063; p-tau181: *p* = 0.68. Figure 1A,B).

We then constructed the density plots of the data distributions, obtained density peaks, and compared the equality of the data distributions. Li-Hep tubes and Na-Hep tubes yielded similar data distribution (tau: K2-EDTA vs Li-Hep, *p* < 0.0001; K2-EDTA vs Na-Hep, *p* = 0.0049; Li-Hep vs Na-Hep, *p* = 0.43; p-tau181: K2-EDTA vs Li-Hep, *p* = 0.05; K2-EDTA vs Na-Hep, *p* = 0.026; Li-Hep vs Na-Hep, *p* = 0.97. Figure 1C,D) and density peaks (tau: K2-EDTA vs Li-Hep vs Na-Hep: 2.81 vs 3.98 vs 4.09; p-tau181: K2-EDTA vs Li-Hep vs Na-Hep: 4.65 vs 5.55 vs 5.43. Figure 1C,D) in plasma tau and p-tau181, both of which were significantly different from K2-EDTA tubes, indicating that different collection tubes affected the distribution of tau and p-tau181 levels in the same cohort.

### 3.3. Blood Collection Methods Do Not Affect the Results of Plasma Aβ

**Figure 2:**
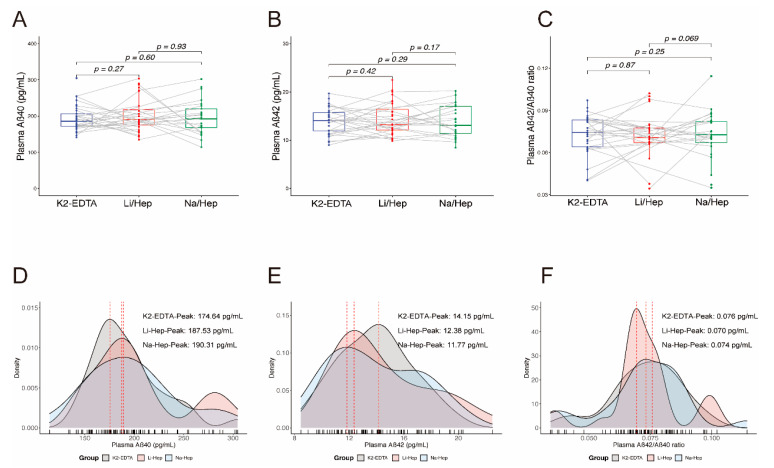
Blood collection methods do not affect the detection of Aβ levels. (**A**–**C**) AD biomarkers comparison among K2-EDTA, Li-Hep, and Na-Hep tubes including Aβ40 (**A**), Aβ42 (**B**), and Aβ42/40 ratio (**C**); (**D**–**F**) Density plot of Aβ40 (**D**), Aβ42 (**E**) and Aβ42/40 ratio (**F**) among K2-EDTA, Li-Hep, and Na-Hep tubes. (**A**–**C**): The differences between groups were assessed by paired t-tests; (**D**–**F**): The peak levels were calculated and statistically compared using the Kolmogorov–Smirnov test. n was indicated in the methods and Table 1.

Plasma Aβ40 and Aβ42 collected in the K2-EDTA tube, Li-Hep tube, and Na-Hep tube were also measured (Figure 2 and Table 1). The mean plasma Aβ40, Aβ42 and their ratio indicate no significant difference in the Li-Hep tube compared with the K2-EDTA tube (Aβ40: K2-EDTA vs Li-Hep: 193.20 ± 35.65 vs 203.20 ± 47.94, *p* = 0.27; Aβ42: K2-EDTA vs Li-Hep: 13.92 ± 2.66 vs 14.41 ± 3.43, *p* = 0.42; Aβ42/40 ratio: K2-EDTA vs Li-Hep: 0.073 ± 0.014 vs 0.073 ± 0.015, *p* = 0.87. Figure 2A–C), or between Na-Hep and K2-EDTA tubes (Aβ40: K2-EDTA vs Na-Hep: 193.20 ± 35.65 vs 197.10 ± 46.04, *p* = 0.60; Aβ42: K2-EDTA vs Na-Hep: 13.92 ± 2.66 vs 13.87 ± 3.28, *p* = 0.29; Aβ42/40 ratio: K2-EDTA vs Na-Hep: 0.073 ± 0.014 vs 0.073 ± 0.017, *p* = 0.25. Figure 2A–C). These were no differences observed between Li-Hep and Na-Hep groups (Aβ40: *p* = 0.93; Aβ42: *p* = 0.17; Aβ42/40 ratio: *p* = 0.069. Figure 2A–C). For density plots of the data distributions of plasma Aβ40, Aβ42 and their ratio among K2-EDTA tube, Li-Hep tube, and Na-Hep tube, there were no apparent patterns, including data distribution (Aβ40: K2-EDTA vs Li-Hep, *p* = 0.60; K2-EDTA vs Na-Hep, *p* = 0.74; Li-Hep vs Na-Hep, *p* = 0.98; Aβ42: K2-EDTA vs Li-Hep, *p* = 0.64; K2-EDTA vs Na-Hep, *p* = 0.85; Li-Hep vs Na-Hep, *p* = 0.86; Aβ42/40 ratio: K2-EDTA vs Li-Hep, *p* = 0.38; K2-EDTA vs Na-Hep, *p* = 0.98; Li-Hep vs Na-Hep, *p* = 0.28. Figure 1D–F) and density peaks (K2-EDTA vs Li-Hep vs Na-Hep, Aβ40: 174.64 vs 187.53 vs 190.31; Aβ42: 14.15 vs 12.38 vs 11.77; Aβ42/40 ratio: 0.070 vs 0.076 vs 0.074. Figure 2D–F), indicating that different collection tubes did not affect the distribution of Aβ40, Aβ42 and their ratio in same cohort.

### 3.4. Blood Collection Methods Does Not Affect the Difference in Biomarkers between Sexes

**Figure 3:**
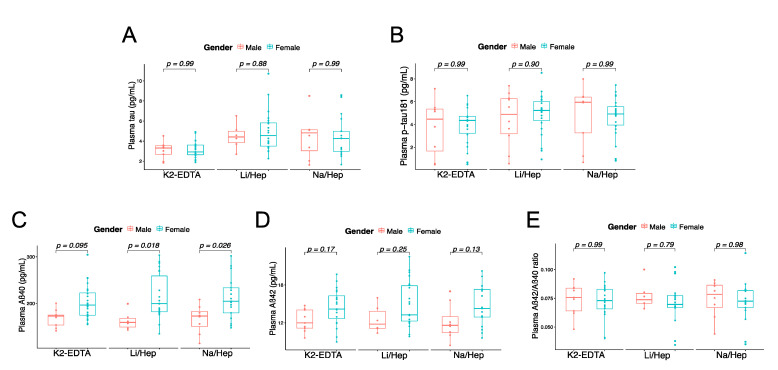
AD biomarkers comparison between genders among K2-EDTA, Li-Hep, and Na-Hep tubes, including tau (**A**), p-tau181 (**B**), Aβ40 (**C**), Aβ42 (**D**), and Aβ42/40 ratio (**E**). The differences between groups were assessed by two-way ANOVA with Sidak multiple comparisons test. n was indicated in the methods and Table 1.

It is known that gender is also a significant risk factor for AD, and we have here analyzed further by dividing our data by gender. We found there were no significant differences in tau (Figure 3A), p-tau181 (Figure 3B), Aβ42 (Figure 3D), and Aβ42/40 ratio (Figure 3E) between males and females across the methods of blood collecting. We have found that plasma Aβ40 levels increased significantly in females in Li-Hep tubes (*p* = 0.018, Figure 3C) and Na-Hep tubes (*p* = 0.026, Figure 3C), and similar results were observed in K2-EDTA tubes (*p* = 0.095, Figure 3C), indicating that blood collection does not affect the difference of biomarkers between genders.

## 4. Discussion

In this study of human samples, we have explored the effects of blood collection tubes on plasma AD biomarkers using the Simoa platform. This method has been widely used in various studies, and as the field moves toward clinical translation, there is a need to understand pre-analytical determinants of measurement values. Meanwhile, Verberk et al. have recently investigated the influences of pre-analytical sample handling for AD blood-based biomarkers, including blood collection tube type, delayed centrifugation time, centrifugation temperature, aliquot volume, delayed storage, and freeze-thawing [23]. Our results were further extended by showing that K2-EDTA tubes under-report tau and p-tau181 values compared to both Li-Hep and Na-Hep tubes, but Aβ40, Aβ42, and Aβ42/Aβ40 ratio analytes were not impacted by blood collecting tubes. Subsequently, AD blood-based biomarkers’ distribution and the differences between males and females were compared among different blood collection tubes, providing further information on AD biomarker studies.

In a survey of the prior literature on plasma tau from Medline, PubMed, Google Scholar, Web of Science, and the Cochrane Library electronic databases, using the keywords’ plasma tau’ and ‘plasma total tau’ during 2013–2022, we identified 65 publications, with only two studies using Li-Hep tubes [18,24]. Also, the value of plasma tau in controls in one article is 2.81 pg/mL, and the value of the other article is 6.24 pg/mL. Therefore, it was unclear whether the measurement of plasma tau was impacted by the blood collecting tubes until the current study.

Since the principle of detection is based on antigen-antibody reactions [25], we speculate that heparin may affect the binding of tau to antibodies during the analysis. Tau is a protein highly enriched in neurons and was initially discovered by its ability to bind and stabilize microtubules [26]. It was reported previously that the aggregation of tau is promoted by heparan sulfate (HS) in vitro [27]. As heparin has a similar chemical property to the more expensive heparan sulfate, it has been used to induce tau aggregation in vitro [28,29,30,31]. We, therefore, propose that heparin in the Li-Hep and Na-Hep tubes may affect the aggregation of plasma tau, which disturbed its binding to the detection antibodies and affected the reading eventually.

## 5. Limitations

There were several limitations in this study. The first is the small sample size of our study. The recruitment of subjects was difficult due to the use of three different blood collection tubes for each subject, however, it could be still considered a good starting point for this type of analysis. The second is that to guarantee that the results were not affected by the disease, age, or education, we selected young healthy adults along with education years of ≥16 years. Therefore, tau and p-tau levels in people who suffer from AD or education years less than 16 years may be influenced by blood collection tubes differently. Meanwhile, only one tau phosphorylation site was detected, and subsequent studies with larger samples and more platforms may be needed.

We also found a higher level of plasma p-tau181 compared to total tau from the same subject. We have analyzed the literature and found that in earlier studies that reported p-tau181 [5,6,32], the average levels of plasma p-tau181 in controls were greater than the average value of plasma tau in meta-analysis [8]. We speculate the results may be due to the manufactural differences in p-tau181 kit batches before 2021, which was also used in the current study.

## 6. Conclusions

This study demonstrated that heparin alters the plasma tau protein values detected by the Simoa method. Therefore, the type of blood collection tubes should be considered when performing studies on plasma tau, and by extension, on biomarker studies of AD.

## Figures and Tables

**Table 1:** Demographic characteristics and AD biomarkers levels of the cohort.

Characteristics	Values
Maximum, n	34
Age, years	28.71 (7.55)
Male, n (%)	11 (32.40)
Education, years	18.38 (1.72)
K2-EDTA-tau, pg/mL	3.15 (0.86)
Li-Hep-tau, pg/mL	4.87 (1.85)
Na-Hep-tau, pg/mL	4.41 (2.02)
K2-EDTA-p-tau181, pg/mL	3.95 (1.86)
Li-Hep-p-tau181, pg/mL	4.84 (1.95)
Na-Hep-p-tau181, pg/mL	4.78 (1.97)
K2-EDTA-Aβ40, pg/mL	193.20 (35.65)
Li-Hep-Aβ40, pg/mL	203.20 (47.94)
Na-Hep-Aβ40, pg/mL	197.10 (46.04)
K2-EDTA- Aβ42, pg/mL	13.92 (2.66)
Li-Hep-Aβ42, pg/mL	14.41 (3.43)
Na-Hep-Aβ42, pg/mL	13.87 (3.28)
K2-EDTA-Aβ42/40 ratio, pg/mL	0.073 (0.014)
Li-Hep-Aβ42/40 ratio, pg/mL	0.073 (0.015)
Na-Hep-Aβ42/40 ratio, pg/mL	0.073 (0.017)

Data are mean (std. deviation) unless otherwise specified.

## Data Availability

The data that support the findings of this study are available from the corresponding author upon reasonable request.
